# "That is why I stopped the ART": Patients' & providers' perspectives on barriers to and enablers of HIV treatment adherence in a South African workplace programme

**DOI:** 10.1186/1471-2458-8-63

**Published:** 2008-02-18

**Authors:** Mison Dahab, Salome Charalambous, Robin Hamilton, Katherine Fielding, Karina Kielmann, Gavin J Churchyard, Alison D Grant

**Affiliations:** 1 Clinical Research Unit, Department of Infectious and Tropical Diseases, London School of Hygiene & Tropical Medicine, London, UK; 2 Aurum Institute for Health Research, Johannesburg, South Africa; 3 Collaborative Programme for AIDS Research in South Africa, University of Kwa-Zulu Natal, Durban, South Africa; 4 Infectious Disease and Epidemiology Unit, Department of Epidemiology and Public Health, London School of Hygiene & Tropical Medicine, London, UK; 5 Health Policy Unit, Department of Public Health Policy, London School of Hygiene & Tropical Medicine, London, UK

## Abstract

**Background:**

As ART programmes in African settings expand beyond the pilot stages, adherence to treatment may become an increasing challenge. This qualitative study examines potential barriers to, and facilitators of, adherence to ART in a workplace programme in South Africa.

**Methods:**

We conducted key informant interviews with 12 participants: six ART patients, five health service providers (HSPs) and one human resources manager.

**Results:**

The main reported barriers were denial of existence of HIV or of one's own positive status, use of traditional medicines, speaking a different language from the HSP, alcohol use, being away from home, perceived severity of side-effects, feeling better on treatment and long waiting times at the clinic. The key facilitators were social support, belief in the value of treatment, belief in the importance of one's own life to the survival of one's family, and the ability to fit ART into daily life schedules.

**Conclusion:**

Given the reported uncertainty about the existence of HIV disease and the use of traditional medicines while on ART, despite a programme emphasising ART counselling, there is a need to find effective ways to support adherence to ART even if the individual does not accept biomedical concepts of HIV disease or decides to use traditional medicines. Additionally, providers should identify ways to minimize barriers in communication with patients with whom they have no common language. Finally, dissatisfaction with clinical services, due to long waiting times, should be addressed.

## Background

The number of HIV-infected individuals receiving ART in Sub-Saharan Africa increased from 100,000 (an estimated 2% of those requiring it) in 2003 to 1.3 million (24–33% of those requiring it) in 2006 [1]. This has given people living with HIV/AIDS greater hope as treatment has led to improved survival and decreased morbidity [2,3].

Though the definition of the "sufficient" level of adherence is still evolving, close adherence to ART remains important to achieving viral suppression, reduced risk of disease progression and better survival [4-6]. Early reports from pilot ART programmes in African settings show high levels of adherence to treatment [7]. However, as ART delivery programmes grow beyond pilot phases, a focus on maintaining these levels is essential. Given that adherence is the strongest predictor of treatment success, a clearer understanding of adherence barriers in low-income countries providing ART is needed [8-11]. Due to the relatively limited experience of ART use in low-income countries, information about barriers to adherence is limited. Even more limited are qualitative studies looking not only at who is having difficulty in adhering but also at why. A recent systematic review of adherence reported on only two such studies [12].

Within the existing body of literature from low-income countries a few factors have more frequently been reported to be associated with poor adherence. These include structural barriers such as high cost and unavailability of drugs [13-15], and individual factors such as lack of self efficacy for medication taking [16,17], lack of social or family support [16,18], high anxiety, perception that ART has a negative effect [16,17], and fear of stigma and discrimination through disclosure of HIV status [19,20]. However, lower level of education has not consistently been reported to be associated with poor adherence [17-19,21].

This study was conducted in a large workplace ART programme providing treatment in South Africa since 2002. All services and medications were provided free of charge to employees with HIV infection. Patients received routine care based on a standardized protocol. All patients underwent intensive adherence counselling aimed at preparing them to start taking ART. Counselling was also offered and provided at all subsequent programme visits. This study mirrored many public sector ART programmes in that it offered ART to all who fulfil medical criteria without other pre-requisites. The purpose of this study was to qualitatively explore patient and health provider perspectives of barriers to, and enablers of, adherence in a workplace setting.

## Methods

### Design and participants

In this qualitative study we held key-informant interviews with two groups of participants. The first group was patients who had received ART for at least 8 weeks. We made a special effort to interview poor adherers. Poor adherers were classified as such if they fulfilled any of the following criteria: reported to clinic staff any difficulties taking treatment regularly and on time; or missed clinic visits; and/or who had less than a 1 log drop in HIV viral load eight weeks after ART start. The second group was individuals involved in, or supporting the provision of treatment and care for ART patients. All health service providers (HSPs) in the HIV wellness clinic and a mine human resources manager were interviewed. A traditional healer was selected in consultation with the traditional healers' association of South Africa, specifically because she worked in close proximity to the wellness clinic. Participants were eligible for the study if they expressed willingness to share their views on adherence to ART and were at least 18 years of age.

### Data collection

Interviews lasting 1 to 1.5 hours were conducted in the language of the participant's choice. All interviews were tape-recorded except two, one because of the participant's preference and the other due to tape recorder malfunction. In both these cases detailed notes were taken. An interpreter facilitated interviews conducted in local languages, transcribed the interviews verbatim and translated them into English. Patients taking ART were interviewed regarding: 1) their experience of ART and how they took it; 2) what helped or kept them from taking ART; 3) what they thought might help or keep other patients from taking ART. Health care providers and the human resources manager were interviewed regarding their perceptions of what helps and/or what keeps patients from taking ART regularly.

### Data analysis

We analysed the interview data using a thematic content analysis approach. This approach is a comparative process by which the various accounts gathered are compared with each other to classify those "themes" that recur or are common in the data set [22]. We perused initial transcripts on a line-by-line basis to identify phrases and assign content-related categories or key themes after multiple readings. Codes were developed to label key themes in the data and to develop a coding scheme. We then analysed the content of each script using the coding scheme to identify the most common or recurrent themes.

### Ethics

Ethics approval for the study was provided by the Biomedical Research Ethics Committee of the University of KwaZulu-Natal (South Africa) and the Ethics Committee of the London School of Hygiene and Tropical Medicine. Individuals willing to participate in the study provided written informed consent for participation.

## Results

The interviews were conducted from June to July 2005 with 12 participants: six ART patients, five health service providers (including one traditional healer), and one company human resources manager. Four of the health care providers were female. The six patients were all male and they had been on ART for more than 8 weeks. Four were classified as poor adherers.

### Key barriers

#### Individual factors

Several individual factors were thought to lead to non-adherence. These were alcohol use, being away from home, fear of stigma, use of traditional medicines, and lack of belief in the existence of HIV and/or one's own status. Finally some participants felt that old age and illiteracy may also lead to poor adherence.

Most participants reported that alcohol use, being away from the usual place of residence and fears of stigma were potential barriers to adherence.

"Alcohol makes you forget things. I used to be taking it then I stopped [taking alcohol]....I used to feel something after taking tables...dizzy. Its alcohol only [that prevents patients from taking ART]." PID 01: Patient

Both patients and health care providers reported that fear of stigmatisation reportedly caused patients who had not disclosed their HIV status to hide their ART or skip doses when in the presence of others.

"Some are scared to disclose ...If a person does not disclose to his partner he will have a problem in taking his medication, because he will have to hide ... and if they have to hide medication ... one day you are not going to take because you'll be scared. oh is she going to see me? Let me not take it today. I'll take it when she has gone." PID 05: HSP

The use of traditional medicines while on ART was also reported as a potential barrier. Both patients and providers felt that ART and traditional medicines should not be "mixed". Some patients who reported using traditional medicines after starting ART also said that they had stopped treatment for the duration of traditional medicine use.

"I tell the patients to take my medicine for one week and then for the other week it must be the tablets [ART] ..." PID 09: Traditional healer

"I had a pain in my foot and the doctors could not help me ... I went to my traditional healer. She gave me something ... That is why I stopped the ART. You shouldn't mix the two!!" PID 06: patient

Providers reported that denial of the existence of HIV or of one's own HIV-positive status kept some patients from taking their treatment.

"Those patients who are not adherent don't believe that HIV is the cause of the illness. ... They believe that they are bewitched or it's got nothing to do with the virus that's causing AIDS". PID 08: provider

A patient who did not think that HIV existed or that he was HIV infected also reported discontinuing treatment because he did not feel that it was always necessary.

"I don't think I have HIV ... Ahh, I am not sure [that HIV exists] ... look at me I am fine ... I am not sick ... I have normal people's sicknesses ... if I am sick again I'll use the best medicine for that sickness, maybe the ART maybe something else." PID 06: patient

Providers believed strongly that older and illiterate patients had more difficulty understanding counselling and instructions regarding how to take treatment and, therefore, had more difficulty in adhering to treatment. However, patients interviewed did not feel that age or educational level would negatively affect their own ability to adhere to treatment.

#### Disease and treatment

Feeling well and experiencing side effects were the main disease and treatment factors reported to influence adherence. Feeling well after taking treatment was reported as both a barrier to, and a facilitator of adherence.

"After I recovered, I noticed that they [ART pills] are working and I continued. I can't stop them" PID 01: poorly adherent patient

"Sometimes when I feel I am right, I don't take them [ART pills]" PID 11: poorly adherent patient

A patient who reported experiencing "severe" side-effects, including hallucinations and insomnia, which affected his ability to work, discontinued ART in the early stages of treatment.

"It [ART] just gave me problem from the first time after using it ... I told them [HSPs] no, I am not going to use them again. I am going to end up not going to work because of them ... That's how I stopped them." PID 03: patient

#### Patient/health care provider relationship

Communication barriers were thought to contribute to poor adherence. Providers felt that this primarily affected patients who spoke languages different from those of their providers.

"... Especially the [cited a specific ethnic group] don't adhere. We try to speak their language ... sometimes he knows a bit of my language, then I talk my language, I ask him if he can hear me." PID 05: provider

#### Health system

Most patients interviewed felt that clinic waiting times were too long. This contributed to their dissatisfaction with clinic services and made them more likely to stop coming to the clinic to pick up their medications.

"We leave the hostels at seven without breakfast ... You can wait again until one o'clock. Then you can see a doctor. You're hungry, you've been waiting, and again you're sick." PID 12: patient

### Key facilitators

The main reported facilitators of adherence were disclosure, having social support and a strong belief in the value of treatment.

However, some patients reported that despite not disclosing their HIV status to their family or friends they still relied on them for support to take treatment.

"I never told them [my family members] that I am taking ART ... I just told them it's my TB treatment ... it was helping because they always reminded me to take my tablets before I go to bed." PID 01: patient

Some patients reported that a strong belief in the value of treatment also helped them adhere to treatment. Additionally the two more adherent patients stressed the importance of their future well-being for the survival of their families and loved ones.

"Nobody reminds me of my medication. I do it on my own, because the only thing I think of if I don't take my treatment is death ... how were they [family members] going to survive if I was dead ... I can't stop them [ART pills]." PID 02: patient

## Discussion

The effect of traditional medicine use on adherence has not been widely reported. In this setting, traditional medicine use appeared to affect adherence negatively. Patients reported that both clinic providers and traditional healers advised them not to "mix" ART with traditional medicines, leading to ART interruptions. Given that traditional medicine use is common in this setting [23], a deeper understanding and clarification to providers of how to counsel patients on the use of traditional medicines while on ART is critical.

A key and, to our knowledge, novel finding of our study is that doubt about the existence of HIV disease, own HIV-infected status, or both were reported to inhibit adherence. In contrast, an earlier study in an urban setting in South Africa indicated that a high proportion of participants had correct knowledge of HIV [12]. This difference in findings perhaps reflects that the earlier study was conducted in an urban setting where traditional beliefs concerning disease causation may be less common.

Results from a pilot quantitative study conducted among 69 patients in this clinic subsequently confirmed that lack of belief in HIV disease or in own status was prevalent. The study showed that 19% patients were uncertain or did not believe that HIV existed, while 24% were uncertain or did not believe that they were HIV-infected [24]. This is despite patients receiving at least three intensive counselling sessions on HIV and ART prior to starting treatment. To what degree this factor truly affects adherence to ART is not yet clear and is being further investigated. Meanwhile, there is a need to find effective ways to support adherence to ART even if the individual does not accept biomedical concepts of HIV disease.

No consistent association has been found in low income countries between level of education and poor adherence [8,25-27]. However, in this setting providers reported that lower levels of education were strongly related to poor adherence, a view not shared by patients interviewed. In a multilingual setting it may be possible that having less education is related to speaking a different language from that of providers, a factor which has previously been found to be related to poor adherence in South Africa [28]. The extent to which education affects adherence in this population, if at all, is not yet clear. However, providers' perceptions that illiterate patients have a poorer ability to adhere may have important implications for how they interact with and provide counselling to patients with less education. This may also be important in other ethnically and linguistically diverse African countries where communication barriers between patients and providers may negatively affect adherence.

Few studies in the existing literature have examined the relationship between adherence and health system factors. Among those that did, cost was found to be the foremost reason for poor adherence [13,14]. This was not a barrier in this setting as a stable and free supply of treatment was available to all patients. However, long waiting times for clinic services were reported as an important barrier to a patient's ability to return to the clinic for service and to pick up medication. This might be especially stigmatizing in this working population as spending additional time in the clinic may require additional time off work. Further research is warranted into health system factors potentially affecting adherence.

Finally, consistent with previously published research in low-income settings, drug side-effects, alcohol use, being away from usual place of residence and fear of stigma were thought to contribute to poor adherence in this setting [19,20]. In contrast, having social support, a strong belief in the value of treatment and the ability to fit ART into daily routines were reported as facilitators of adherence [16,18]. Interestingly, most patients, even those who were not completely adherent, reported having some social support and/or belief in the value of treatment. However, these alone did not seem to ensure complete adherence. This suggests that in this setting careful consideration of multifaceted interventions to promoting adherence is important.

In our study we purposively sampled a relatively small number of individuals who were not intended to be representative of the clinic population, and our results may not be generalisable. The study was designed to identify potential barriers to adherence relevant to this setting, using in-depth interviews and taking various perspectives into account. Specifically, by complementing patient information with provider information we were able to describe a more complete picture of adherence issues. There was great congruency between factors mentioned by the patients and by the providers, to the extent that after interviewing 12 participants, we felt we had reached saturation of findings. We are currently conducting a much larger quantitative study that investigates the prevalence of these barriers in the population, and their association with poor adherence. However further work is needed to establish whether these factors are also associated with poor adherence in other populations The study setting, among mostly male participants involved in a workplace ART programme, may also limit generalisability. However issues related to uncertainty about western biomedical concepts may arise in other populations, particularly those where traditional, non-western concepts concerning illness are prevalent.

Given the above limitations, our data highlight novel barriers to adherence that merit further study in populations where traditional beliefs around disease causation are common. Our data also illustrate the value of formative qualitative research in identifying culturally-relevant barriers to adherence, particularly given the paucity of such studies in low-income countries [12].

## Conclusion

Uncertainty about the existence of HIV disease and use of traditional medicines while on ART was reported despite a programme emphasising ART counselling. Thus there is a need to find effective ways to support adherence to ART even if the individual does not accept biomedical concepts of HIV disease or decides to use traditional medicines. Additionally, providers should identify ways to minimize barriers in communication with patients who speak different languages. Finally, dissatisfaction with clinical services, namely due to long waiting times, should be addressed.

## Competing interests

The author(s) declare that they have no competing interests.

## Authors' contributions

MD: Developed protocol, coordinated data collection, conducted data analysis and drafted the manuscript. SC: Participated in study design, analysis and supervised data collection. RH: participated in study design and analysis. KF: Advised on study design and analysis. KK: Advised on study design and analysis. GJC: Advised on study design and analysis. ADG: Supervised study design, data collection, and analysis. All authors participated in critical appraisal and revision of the manuscript. All authors have given final approval of the version to be published.

## Pre-publication history

The pre-publication history for this paper can be accessed here:


